# Bacteriological and molecular characterization of temperature- and CO_2_-dependent *Streptococcus pneumoniae* serotype 24F ST162 isolated from Japanese children

**DOI:** 10.1128/spectrum.02165-23

**Published:** 2023-10-12

**Authors:** Jun Kobayashi, Misako Ohkusu, Takehisa Matsumoto, Noriko Kubota, Naruhiko Ishiwada

**Affiliations:** 1 Department of Laboratory Medicine, Nagano Children’s Hospital, Azumino, Japan; 2 Life Science Research Center, Nagano Children’s Hospital, Azumino, Japan; 3 Department of Infectious Diseases, Medical Mycology Research Center, Chiba University, Chiba, Japan; 4 Department of Laboratory Sciences, Gunma University Graduate School of Health Sciences, Maebashi, Japan; Griffith University, Southport, Gold Coast, Queensland, Australia

**Keywords:** *Streptococcus pneumoniae*, MurF, CO_2_-dependent, serotype 24F, ST162

## Abstract

*Streptococcus pneumoniae* serotype 24F is one of the most prevalent non-vaccine serotypes that causes invasive pneumococcal disease (IPD) in many countries, including Japan. This study aimed to analyze the bacteriological and molecular characteristics of serotype 24F sequence type (ST) 162, which has been increasingly isolated from pediatric patients with IPD in Japan recently. The examination of growth conditions, sequencing of genes associated with the CO_2_ dependence, and antimicrobial susceptibility testing at 35°C under 5% CO_2_ were performed for 10 isolates obtained from Japanese children with IPD caused by serotype 24F ST162. All isolates failed to grow at 35°C in ambient air; however, they showed growth at 30°C in ambient air and at 35°C under 5% CO_2_. Sequencing of *murF* involved in cell wall synthesis indicated that all isolates had a single amino acid substitution, MurF^A179V^. Additionally, all isolates were sensitive to penicillin G and resistant to trimethoprim-sulfamethoxazole. Furthermore, 25 non-capnophilic strains were obtained from all CO_2_-dependent isolates, and their *murF* sequences were compared. Thirteen of the 25 non-capnophilic strains displayed a different amino acid substitution, MurF^V179A^, whereas the other 12 presented the previously described MurF^A179V^. This suggests that a proportion of the CO_2_-dependent phenotype of serotype 24F ST162 may have been conferred by MurF^A179V^; however, the mechanism for the CO_2_ dependence of these isolates warrants further investigation. The CO_2_-dependent serotype 24F ST162 pneumococcal isolates shared common characteristics in terms of growth patterns, molecular basis, and antimicrobial susceptibility; therefore, future epidemiological trends of this clone must be closely monitored.

**IMPORTANCE:**

We characterized *Streptococcus pneumoniae* serotype 24F sequence type (ST) 162 isolated from Japanese children with invasive pneumococcal disease (IPD). Owing to its highly invasive nature, serotype 24F is expected to be isolated from clinically significant cases. Serotype 24F ST162 isolates tested in the present study did not grow at 35°C in ambient air. Therefore, antimicrobial susceptibility testing using the broth microdilution method, which is usually conducted in ambient air, cannot be performed, posing a clinical challenge. Clinical practitioners and laboratory personnel should be aware of the epidemiological, bacteriological, and molecular characteristics of serotype 24F ST162. We believe that our findings can help diagnose and treat IPD caused by serotype 24F ST162, a serotype expected to become problematic in the post-13 valent pneumococcal conjugate vaccine era.

## INTRODUCTION

*Streptococcus pneumoniae* is a common cause of pneumonia and acute otitis media in children, who often carry this bacterium in their nasopharynx. *S. pneumoniae* can also cause invasive pneumococcal diseases (IPDs), such as meningitis and sepsis, which have a substantially high mortality rate (1). The major virulence factor of *S. pneumoniae* is an antiphagocytic polysaccharide capsule. A heptavalent pneumococcal conjugate vaccine (PCV7) containing serotypes 4, 6B, 9V, 14, 18C, 19F, and 23F was developed to prevent IPD in children. In Japan, PCV7 was introduced as a voluntary vaccine for children under 5 years of age from October 2010 and has since been included in the routine vaccination schedule. PCV7 was replaced by 13-valent PCV (PCV13) that contains six additional serotypes (1, 3, 5, 6A, 7F, and 19A) for routine vaccination in November 2013 (2). With the widespread use of PCV13, the incidence of IPD caused by PCV13 serotypes has substantially decreased in Japanese children; however, the prevalence of IPD caused by some non-vaccine serotypes (NVTs), such as 10A, 12F, 15A, 15B, 15C, 22F, 24F, 33F, and 35B, has increased (3). Serotype 24F is one of the most common serotypes associated with IPD following the introduction of PCV13 in Japan (3, 4) and other countries (5 – 7). Serotype 24F has been detected in cases of bacteremia among children under 2 years of age at a significantly higher rate than that seen in older children in Japan (8).

*S. pneumoniae* can generally grow at 35°C either in ambient air or 5% CO_2_, but approximately 5%–10% of all pneumococcal isolates are capnophiles requiring CO_2_-enriched growth conditions (9, 10). Burghout et al. reported that the inactivation of pneumococcal carbonic anhydrase (PCA) (11) or dihydrofolate: folylpolyglutamate synthetase (FolC) (12) and a single amino acid substitution in MurF (13) are associated with CO_2_-dependent growth of *S. pneumoniae*. Notably, CO_2_-dependent growth restriction has only been observed in some specific isolates, with Austrian et al. reporting in 1966 that serotypes 1, 3, 16, 28, and 33 accounted for 70% of CO_2_-dependent isolates (9). Additionally, Burghout et al. reported in 2013 that serotypes 9V and 19F were the main serotypes of CO_2_-dependent isolates associated with a single amino acid substitution in MurF, MurF^A179V^ (13). Despite typically performing antimicrobial susceptibility testing of *S. pneumoniae* using the broth microdilution method in ambient air, obtaining optimal results for CO_2_-dependent isolates is challenging.

We have recently identified several serotype 24F sequence type (ST) 162 pneumococcal isolates in Japanese children with IPD, all of which were CO_2_-dependent. Studies on the CO_2_ dependence of *S. pneumoniae* are scarce, and neither the epidemiology nor the mechanisms of CO_2_-dependent *S. pneumoniae* have been reported, especially from Japan. Moreover, there are no reports on any CO_2_-dependent isolates of serotype 24F. Therefore, we analyzed clinical isolates using several methods to clarify the bacteriological and molecular biological characteristics of serotype 24F ST162, which has been recently presumed to be an increasingly prevalent causative agent of pediatric IPD in Japan. We believe that our findings could aid in diagnosing and treating IPD caused by serotype 24F strains that will become a problem in the post-PCV13 era.

## RESULTS

### Clinical features of IPD cases with serotype 24F ST162 and characteristics of isolates

A summary of the 10 IPD cases caused by serotype 24F ST162 is presented in Table 1. The median age of the patients was 2 years (range, 6 months–7 years). Four and six patients were boys and girls, respectively. Five patients had bacteremia without focus, two had meningitis, one had pneumonia with bacteremia, one had cellulitis with bacteremia, and one had arthritis. The isolation materials were blood, cerebrospinal fluid, and joint fluid in eight, one, and one case(s), respectively.

**TABLE 1:** Summary of the CO_2_-dependent pneumococcal isolates and non-capnophilic strains obtained by subculturing the parent isolate^*b*^

CO_2_-dependent isolates												Noncapnophilic strains obtained by subculturing the parent isolate			
No.	Isolate	Age	Sex	Diagnosis	Isolation site	Isolation place	Isolation year	Serotype	MLST	GPSC	MurF 179	Strain no.	Number of subcultures^*a*^	MurF 179	Mutation in upstream of murFcompared to parent isolate
1	17 P48	1year	Female	Pneumonia	Blood	Nagasaki	2017	24F	162	N/A	Val	17P48-nc1	5th	Ala	N.D
												17P48-nc2	6th	Val	N.D
												17P48-nc3	6th	Val	T to G (−16 bp)
2	18 P172	7year	Female	Bacteremia	Blood	Tokyo	2018	24F	162	N/A	Val	18P172-nc1	4th	Ala	N.D
												18P172-nc2	3rd	Ala	N.D
												18P172-nc3	5th	Ala	N.D
												18P172-nc4	5th	Ala	N.D
3	19 P33	6 months	Female	Meningitis	CSF	Ohita	2019	24F	162	N/A	Val	19P33-nc1	4th	Ala	N.D
												19P33-nc2	4th	Ala	N.D
												19P33-nc3	4th	Ala	N.D
4	19 P71	2year	Female	Bacteremia	Blood	Tokyo	2019	24F	162	N/A	Val	19P71-nc1	5th	Ala	N.D
												19P71-nc2	6th	Val	N.D
												19P71-nc3	6th	Val	N.D
5	20 P4	1year	Female	Meningitis	Blood	Tokyo	2020	24F	162	N/A	Val	20P4-nc1	5th	Ala	N.D
												20P4-nc2	5th	Ala	N.D
6	20 P18	1year	Female	Celulitis	Blood	Chiba	2020	24F	162	N/A	Val	20P18-nc1	7th	Val	T to C (−15 bp)
												20P18-nc2	7th	Val	T to C (−15 bp)
7	20 P23	2year	Male	Bacteremia	Blood	Tokyo	2020	24F	162	N/A	Val	20P23-nc1	7th	Val	N.D
												20P23-nc2	7th	Ala	N.D
8	21 P19	2year	Male	Bacteremia	Blood	Tokyo	2021	24F	162	6	Val	21P19-nc1	2nd	Val	N.D
9	21 P23	1year	Male	Arthritis	Joint fluid	Chiba	2021	24F	162	N/A	Val	21P23-nc1	6th	Val	N.D
10	21 P20	3year	Male	Bacteremia	Blood	Nagano	2021	24F	162	6	Val	21P20-nc1	3rd	Val	C to A (−20 bp)
												21P20-nc2	4th	Val	A to T (−75 bp)
												21P20-nc3	2nd	Val	C to A (−20 bp)
												21P20-nc4	2nd	Ala	N.D

^*a*^ Number of subcultures of parental isolates until non-capnophilic strains are obtained.

^*b*^ CSF, Cerebrospinal fluid; GPSC, Global pneumococcal sequencing cluster; MLST, Multi locus sequencing typing; N/A, Not available; N.D, not detected

All isolates showed a CO_2_-dependent phenotype and belonged to serotype 24F ST162. The whole-genome sequencing (WGS) data were available for 21P19 and 21P20, which were identified as belonging to Global Pneumococcal Sequence Cluster (GPSC) 6. Furthermore, the non-capnophilic strains were obtained by subculturing each of the CO_2_-dependent isolates. The list of non-capnophilic strains and the number of subcultures until non-capnophilic strains were obtained are shown in Table 1.

### Growth conditions

**Fig 1:**
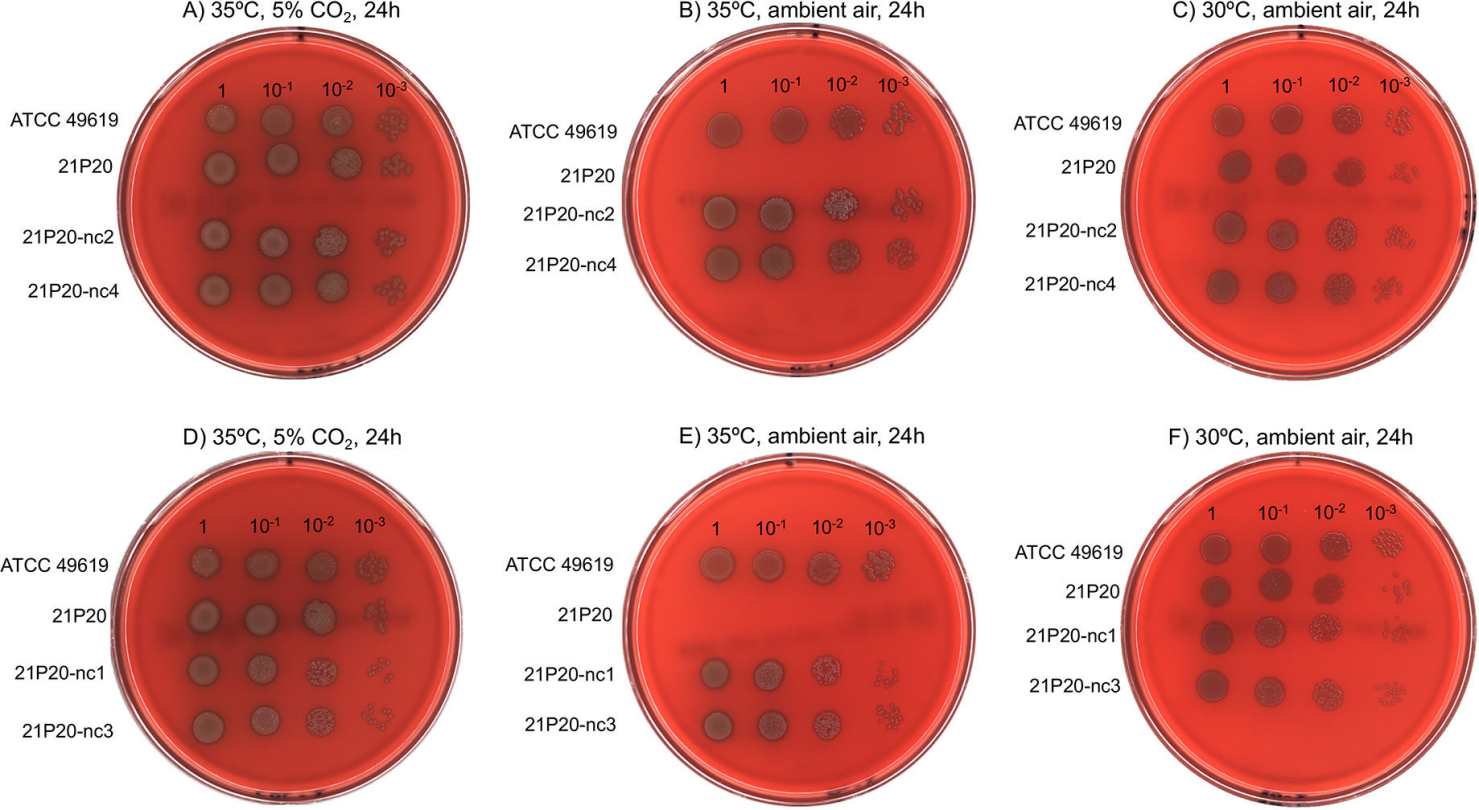
Growth conditions of 21P20, a CO_2_-dependent serotype 24F ST162 isolate, and 21P20-nc2, 21P20-nc4, 21P20-nc1, and 21P20-nc3, four non-capnophilic strains obtained by subculturing 21P20. ATCC 49619 strain was used as a control. The strains were incubated on trypticase soy agar with 5% sheep blood for 24 h (**A, D**) at 35°C under 5% CO_2_, (**B, E**) at 35°C in ambient air, and (**C, F**) at 30°C in ambient air. The CO_2_-dependent isolate 21P20 could not grow at 35°C in ambient air but successfully grew at 30°C in ambient air and at 35℃ under 5% CO_2_. In contrast, the non-capnophilic strains 21P20-nc2 and 21P20-nc4 were able to grow (**A**) at 35°C under 5% CO_2_, (**B**) at 35°C in ambient air, and (**C**) at 30°C in ambient air. Similarly, the non-capnophilic strains 21P20-nc1 and 21P20-nc3 could grow (**D**) at 35°C under 5% CO_2_, (**E**) at 35°C in ambient air, and (**F**) at 30°C in ambient air.

All isolates failed to grow after 24 h of incubation at 35°C in ambient air. In contrast, they successfully grew after 24 h of incubation at 30°C in ambient air and 35°C under 5% CO_2_. The growth condition results for 21P20 as a representative CO_2_-dependent isolate are shown in Fig. 1.

In addition, non-capnophilic strains obtained by subculturing each of the CO_2_-dependent isolates were also examined for growth conditions. All non-capnophilic strains were capable of growing at 35°C under 5% CO_2_, 35°C in ambient air, and 30°C in ambient air. The growth condition results for representative non-capnophilic strains 21P20-nc2 and 21P20-nc4 are shown in Fig. 1A through C, and 21P20-nc1 and 21P20-nc3 in Fig. 1D through F.

### Sequencing of *murF*, *pca*, and *folC*

**Fig 2:**
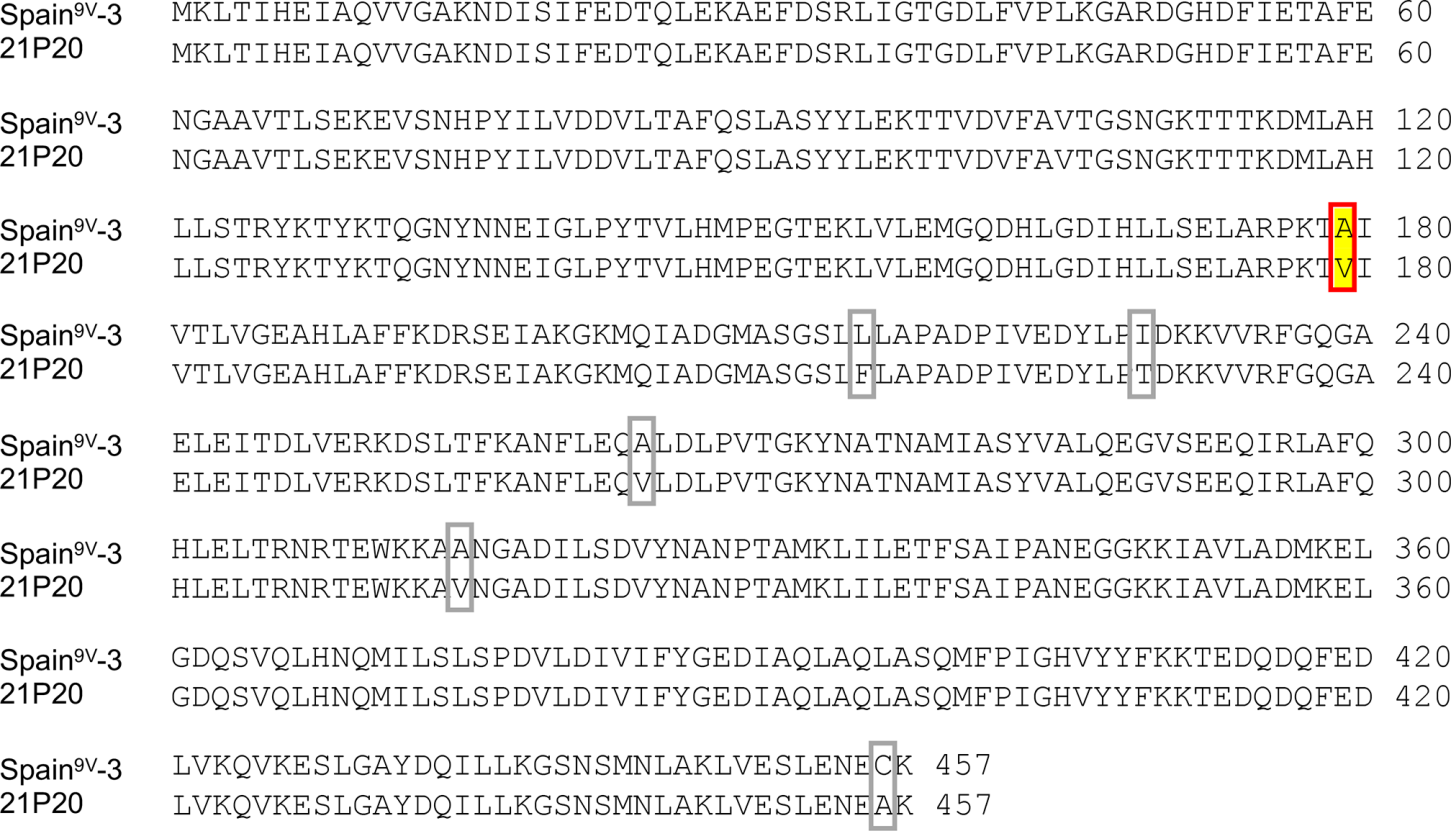
Comparison of amino acid sequence of MurF between 21P20, a representative CO_2_-dependent isolate, and Spain^9V^-3, a reference. Six amino acid substitutions were detected in the 21P20. Among these amino acid substitutions, Val-179 has been suggested to be related to the temperature- and CO_2_-dependent phenotype of *S. pneumoniae* (13). The position is highlighted in yellow and boxed by a solid line in red.

Sequencing of *murF* revealed that all 10 CO_2_-dependent isolates had a valine at position 179 of MurF (Table 1; Fig. 2). In contrast, Spain^9V^-3, a non-capnophile and the reference strain of the pneumococcal molecular epidemiology network (PMEN) collection of clonal complex 156, to which the ST 162 strain belongs, has an alanine at position 179 of MurF (13). All tested CO_2_-dependent isolates differed from Spain^9V^-3 in their amino acid sequence at five positions, Phe-215, Thr-229, Val-264, Val-315, and Ala-456 in addition to position 179 (Fig. 2). The amino acid sequences of PCA and FolC of all tested isolates were identical to those of Spain^9V^-3.

### Antimicrobial susceptibility

Antimicrobial susceptibility results are presented in Table 2. All 10 CO_2_-dependent isolates showed low minimal inhibitory concentrations (MICs) for penicillin G, cefotaxime, ceftriaxone, imipenem, levofloxacin, vancomycin, and chloramphenicol. However, all 10 CO_2_-dependent isolates exhibited resistance to trimethoprim-sulfamethoxazole. Only 17P48, isolated in 2017, was resistant to clarithromycin and clindamycin. Antimicrobial susceptibility results performed in ambient air for the non-capnophilic strains 21P19-nc1 and 21P20-nc1 are also shown in Table 2. The antimicrobial susceptibility patterns of CO_2_-dependent isolate 21P19 and 21P19-derived non-capnophilic strain 21P19-nc1 were generally identical. Similarly, the interpretive categories for all antimicrobial agents were consistent for both 21P19 and 21P19-nc1 although MICs for clarithromycin and clindamycin tended to be higher for the former. The same tendencies were observed in the comparison of the CO_2_-dependent isolate 21P20 with the non-capnophilic strain 21P20-nc1 derived from 21P20.

**TABLE 2:** Antimicrobial susceptibility of pneumococcal isolates and ATCC 49619 used as a quality control^*d*^

Strain no.	Testing	MIC (µg/mL)									
	Conditions	PCG	CTX	CTRX	IPM	CAM	CLDM	LVFX	VCM	CP	SXT
17P48	35°C, 5% CO_2_	≤0.06 (S)	0.25 (S)	0.12 (S)	≤0.06 (S)	>8 (R)	>8 (R)	1 (S)	0.5 (S)	4 (S)	>4/76 (R)
18P172	35°C, 5% CO_2_	≤0.06 (S)	≤0.06 (S)	≤0.06 (S)	≤0.06 (S)	0.12 (S)	0.25 (S)	2 (S)	0.5 (S)	2 (S)	>4/76 (R)
19P33^*a*^	35°C, 5% CO_2_	≤0.06 (S)	≤0.06 (S)	≤0.06 (S)	≤0.06	0.12	0.25	1	0.5 (S)	2	>4/76
19P71	35°C, 5% CO_2_	≤0.06 (S)	≤0.06 (S)	≤0.06 (S)	≤0.06 (S)	0.12 (S)	0.25 (S)	1 (S)	0.5 (S)	2 (S)	>4/76 (R)
20P4	35°C, 5% CO_2_	≤0.06 (S)	≤0.06 (S)	≤0.06 (S)	≤0.06 (S)	0.12 (S)	0.25 (S)	1 (S)	0.5 (S)	2 (S)	>4/76 (R)
20P18	35°C, 5% CO_2_	≤0.06 (S)	≤0.06 (S)	≤0.06 (S)	≤0.06 (S)	0.12 (S)	0.25 (S)	1 (S)	0.25 (S)	2 (S)	>4/76 (R)
20P23	35°C, 5% CO_2_	≤0.06 (S)	≤0.06 (S)	≤0.06 (S)	≤0.06 (S)	≤0.06 (S)	0.25 (S)	1 (S)	0.5 (S)	2 (S)	>4/76 (R)
21P19	35°C, 5% CO_2_	≤0.06 (S)	≤0.06 (S)	≤0.06 (S)	≤0.06 (S)	0.12 (S)	0.25 (S)	1 (S)	0.5 (S)	2 (S)	>4/76 (R)
21P19-nc1^*b*^	35°C, ambient air	≤0.06 (S)	≤0.06 (S)	≤0.06 (S)	≤0.06 (S)	≤0.06 (S)	0.12 (S)	1 (S)	0.5 (S)	2 (S)	>4/76 (R)
21P23	35°C 5% CO_2_	≤0.06 (S)	≤0.06 (S)	≤0.06 (S)	≤0.06 (S)	≤0.06 (S)	0.25 (S)	1 (S)	0.5 (S)	2 (S)	>4/76 (R)
21P20	35°C, 5% CO_2_	≤0.06 (S)	≤0.06 (S)	≤0.06 (S)	≤0.06 (S)	0.12 (S)	0.25 (S)	1 (S)	0.5 (S)	2 (S)	>4/76 (R)
21P20-nc1^*c*^	35°C, ambient air	≤0.06 (S)	≤0.06 (S)	≤0.06 (S)	≤0.06 (S)	≤0.06 (S)	≤0.06 (S)	2 (S)	0.5 (S)	2 (S)	>4/76 (R)
ATCC 49619	35°C, 5% CO_2_	0.25	0.12	0.12	<0.06	0.12	0.25	2	0.5	4	2/38
ATCC 49619	35°C, ambient air	0.25	0.12	<0.06	<0.06	<0.06	0.12	1	0.25	2	1/19
MIC QC ranges	35°C, ambient air	0.25–1	0.03–0.12	0.03–0.12	0.03–0.12	0.03–0.12	0.03–0.12	0.5–2	0.12–0.5	2–8	0.12/2.4–1/19

^*a*^ Meningitis interpretive categories of only PCG, CTX, CTRX, VCM is shown because of the strain isolated from cerebrospinal fluid.

^*b*^ Non-capnophilic strain obtained by subculturing 21P19, a CO_2_-dependent isolate.

^*c*^ Non-capnophilic strain obtained by subculturing 21P20, a CO_2_-dependent isolate.

^*d*^ MIC, minimum inhibitory concentrations; PCG, penicillin G; CTX, cefotaxime; CTRX, ceftriaxone; IPM, imipenem; CAM, clarithromycin; CLDM, clindamycin; LVFX, levofloxacin; VCM, vancomycin; CP, chloramphenicol; SXT, trimethoprim-sulfamethoxazole; QC, quality control.

The MICs of each antimicrobial agent against ATCC 49619, used as a quality control strain, are shown in Table 2. All test results, performed at 35°C in ambient air according to Clinical and Laboratory Standards Institute (CLSI) guidelines (14), met the criteria for quality control set forth by CLSI. However, the MICs of clindamycin and trimethoprim-sulfamethoxazole against ATCC 49619 at 35°C under 5% CO_2_ were higher than the CLSI quality control range at 35°C in ambient air.

### Comparison of the genome sequences of CO_2_-dependent isolates and non-capnophilic strains

**Fig 3:**
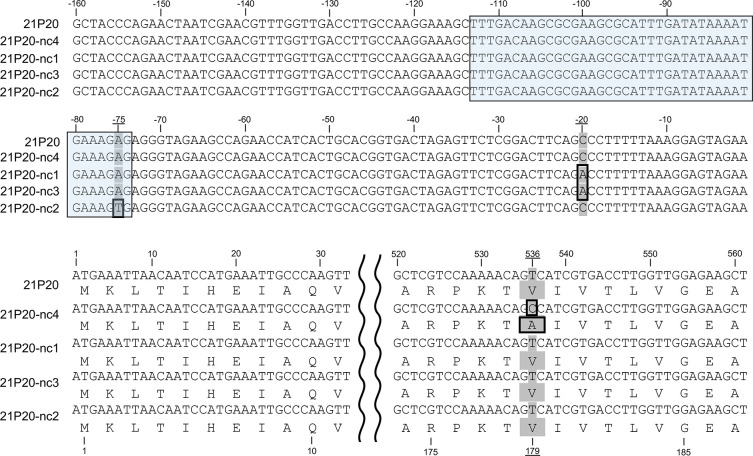
Comparison of *murF* and its upstream sequences in the CO_2_-dependent isolate 21P20 and the non-capnophilic strains 21P20-nc4, 21P20-nc1, 21P20-nc3, and 21P20-nc2. Nucleotide numbers relative to the translational start site are denoted above the nucleotides, and amino acid numbers counted from the amino-terminal methionine are mentioned below the amino acids. The non-capnophilic strain 21P20-nc4 had a single-nucleotide substitution causing a single amino acid substitution in MurF, changing valine to alanine at position 179. The other non-capnophilic strains 21P20-nc1, 21P20-nc3, and 21P20-nc2 had a single-nucleotide substitution upstream of *murF*. The promoter region predicted by Promotech is shaded with a light blue background. 21P20-nc2 had a single-nucleotide substitution at the 3′ end of the sequence predicted to be this promoter region.

WGS was performed on 21P19, a CO_2_-dependent isolate, and 21P19-nc1, a non-capnophilic strain derived from 21P19, and on 21P20, a CO_2_-dependent isolate, and 21P20-nc1, 21P20-nc2, 21P20-nc3, 21P20-nc4, non-capnophilic strains derived from 21P20 to compare the nucleotide sequence in each non-capnophilic strains and their parent isolates. A comparison of 21P20 and 21P20-nc4 revealed that 21P20 has a valine at position 179 in MurF, whereas 21P20-nc4 has an amino acid alteration to alanine similar to that in other non-capnophilic strains such as Spain^9V^-3 (Fig. 3). The percentage of reads with the single-nucleotide polymorphism (SNP) change in 21P20-nc4 was 99.3% (Table 3). In a comparison between 21P20 and 21P20-nc4 by Snippy, there were no amino acid substitutions detected except for position 179 in MurF. In contrast, the other three non-capnophilic strains, 21P20-nc1, 21P20-nc2, and 21P20-nc3, had a valine at position 179 of MurF similar to CO_2_-dependent isolate 21P20. However, the minus 20th base of the *murF* sequence was changed from C to A in 21P20-nc1 and 21P20-nc3, and the minus 75th base of the *murF* sequence was changed from A to T in 21P20-nc2 (Fig. 3). The single-nucleotide substitution upstream of *murF* found in non-capnophilic strain 21P20-nc2 was located within the promoter region predicted by Promotech (Fig. 3). The percentage of reads with the SNP change in 21P20-nc1, 21P20-nc3, and 21P20-nc2 were 99.6%, 99.3%, and 100%, respectively (Table 3). Furthermore, 21P20-nc1 and 21P20-nc3 had single-nucleotide substitutions or frameshift mutations in other genes (Table S1).

**TABLE 3:** Percentage of reads with the single-nucleotide polymorphism change associated with *murF* in non-capnophilic strains obtained from 21P20

Strains	Position	Reference	Alteration		Description
		Base	Base	Percentage	
21P20-nc4	1547401	T	C	159/160 (99.3%)	MurF c.T536C: p.Val179Ala
21P20-nc1	1547956	C	A	519/521 (99.6%)	20-bp upstream of the start codon of *murF*
21P20-nc3	1547956	C	A	295/297 (99.3%)	20-bp upstream of the start codon of *murF*
21P20-nc2	1548011	A	T	454/454 (100%)	75-bp upstream of the start codon of *murF*

Compared to the parent isolate, the non-capnophilic strain 21P19-nc1, obtained from another CO_2_-dependent isolate 21P19, harbored nucleotide substitutions related to hypothetical proteins and transposases; however, *murF* and its upstream and downstream sequences were unaltered (Table S2).

For non-capnophilic strains other than those mentioned above, *murF* sequences were determined using Sanger sequencing and compared with the sequences of their respective parental isolates (Table 1). Collectively, the WGS and Sanger sequencing results showed that 13 of the 25 non-capnophilic strains had a substitution of amino acid 179 in MurF from valine to alanine, whereas the remaining 12 strains continued to contain valine. Additionally, 6 of the 12 strains had a single-nucleotide substitution upstream of *murF*.

## DISCUSSION

In this study, we characterized *S. pneumoniae* serotype 24F ST162 isolated from pediatric patients with IPD in Japan and identified several common features. All 10 isolates were CO_2_-dependent when incubated at 35°C, whereas they could grow at 30°C in ambient air. Sequencing of *murF* demonstrated that all isolates had valine at position 179 of MurF. As some of the non-capnophilic strains obtained from CO_2_-dependent isolates contained valine at position 179 of MurF, this amino acid substitution cannot be entirely explained as the cause of the CO_2_-dependent phenotype; however, approximately half of the non-capnophilic strains had the 179th amino acid of MurF changed from valine to alanine, suggesting that MurF^V179^ may partially explain the temperature and CO_2_ dependence of the isolates in this study. Furthermore, antimicrobial susceptibility testing revealed that all tested isolates were susceptible to penicillin G but resistant to trimethoprim-sulfamethoxazole. Collectively, this study is the first to demonstrate that CO_2_-dependent *S. pneumoniae* isolated from Japanese children with IPD are associated with a specific serotype and ST, namely, serotype 24F ST162 that has a single amino acid substitution at position 179 of MurF and exhibits a common antimicrobial sensitivity pattern, such as susceptibility to penicillin G and resistance to trimethoprim-sulfamethoxazole.

Strains with serotype 24F, an NVT, have been increasingly isolated from IPD patients since the introduction of PCV13 in Japan and other countries such as France (5), Denmark (7), Norway (6), England, and Wales (15). Serotype 24F has been associated with ST162 in France (5), Denmark (7), and Norway (6). In Asia, a case of severe meningitis owing to serotype 24F ST162 strain has been reported from Hong Kong (16); however, none of the reports mentioned the CO_2_ dependence of the strains. According to surveillance data from 2017 in Japan, despite the high incidence of IPD caused by serotype 24F, ST162 was not included (3). ST2572 and ST5496 (single-locus variants of ST2572), which primarily include serotype 24F and are susceptible to penicillin, spread significantly between 2012 and 2014 in Japan (4). This trend was also observed in the surveillance data between 2015 and 2017 (17); however, serotype 24F ST162 was not detected. In contrast, the most recent epidemiological analysis of pediatric IPD in a Japanese prefecture detected one ST162 strain of serotype 24F in the period 2010–2020 (8). The increase in the number of isolates of this clone in this study from 2017 to 2021 suggests an increase in the number of serotype 24F ST162 strains in Japan, especially in the last few years. Thus, continued monitoring of this clone will be necessary in the future. In 2021, a novel PCV20 vaccine that covered seven additional serotypes (8, 10A, 11A, 12F, 15B, 22F, and 33F) in addition to those included in PCV13 was licensed by the Food and Drug Administration for adults aged ≥18 years (18). Trials on the safety and efficacy of PCV20 in infants are underway in the United States to promote its clinical application (19). However, PCV20 does not cover serotype 24F; PCV21, which is under development, covers serotype 24F but is for adults (20). Therefore, it is necessary to develop a new PCV vaccine that also covers serotype 24F for children. However, there is also concern regarding serotype replacement for other NVTs. To address these problems, research and development of non-capsular polysaccharide-based vaccines have become active (21). However, the elimination of all pneumococcal colonization by non-capsular polysaccharide-based vaccines may not be advisable (22) because there are reports that pneumococcal colonization on the nasopharynx is beneficial to the host from an immunological perspective (23, 24), and there are concerns regarding the vaccine’s influence on the composition of human nasopharyngeal flora (25). Therefore, it is essential to develop a better pediatric vaccine program that takes all these concerns into account.

All isolates with serotype 24F ST162 analyzed in this study were CO_2_-dependent. Notably, these isolates were CO_2_-dependent at 35°C but could grow at 30°C in ambient air. We analyzed the sequences of PCA and FolC, which have been reported to be associated with CO_2_-dependent *S. pneumoniae*. Compared with the related PMEN clone Spain^9V^-3, the isolates analyzed in this study revealed no changes in their amino acid sequences. Therefore, we concluded that PCA and FolC were unrelated to the CO_2_ dependence of the pneumococcal isolates in this study. In contrast, sequencing of *murF* revealed that all isolates had six amino acid substitutions, Val-179, Phe-215, Thr-229, Val-264, Val-315, Ala-456, in MurF (Fig. 2). Burghout et al. (13) reported that the introduction of MurF^V179^ into a CO_2_-independent strain imposed capnophilic growth restriction, and changing the 179th valine of MurF to alanine resulted in the elimination of capnophilic growth restriction; however, MurF Thr-229, Val-264, and Ala-456 are presumably not associated with the CO_2_-dependent phenotype because Thr-229 and Val-264 are also present in non-capnophilic R6, TIGR4, and G54 strains, and the 456th amino acid is also variable in those strains. In addition, Burghout et al. created mutants for Val-179, Phe-215, and Val-315 by mutating them to Ala-179, Leu-215, and Ala-315, respectively, which Spain^9V^-3 already possessed, and introduced MurF^V179A^, MurF^F215L^, and MurF^V315A^ into the Spain^9V^-3 strain. They concluded that the presence of valine at position 179 in MurF, rather than the presence of Phe-215 and Val-315, renders the CO_2_-dependent phenotype because the yield of the non-capnophilic mutant with MurF^V179A^ was comparable to that of Spain^9V^-3, but MurF^F215L^ and MurF^V315A^ had lower yields (13). The *S. pneumoniae* with valine at position 179 of MurF does not grow at 34°C and 37°C in ambient air but can grow at 30°C (13). The mechanisms underlying the unique growth conditions of *S. pneumoniae* with MurF^V179^ are not fully understood; nonetheless, both temperature-induced changes in MurF stability and the carbamoylation state of the Lys-202 residue at low environmental CO_2_ concentrations may be related to the growth conditions of this strain (13). We speculated that it failed to grow at 35°C in ambient air because of the structural dysfunction of MurF involved in the final stage of peptidoglycan synthesis. However, this hypothesis may not be sufficient to explain the CO_2_-dependent mechanism of isolates tested in this study. WGS data for selected strains and Sanger sequencing of *murF* for the other strains showed that 13 of the 25 non-capnophilic strains changed to alanine at position 179 of MurF, whereas the parent strains had valine, and that the non-capnophilic strains could grow at 35°C in ambient air. In contrast, 6 of 25 non-capnophilic strains retained MurF^V179^ but had a single-nucleotide substitution upstream of the *murF* sequence. We hypothesized that if the site was a promoter- or repressor-binding sequence, the increased expression of MurF in these non-capnophilic strains might have compensated for the reduced activity of MurF^V179^ and facilitated their growth at 35°C in ambient air. In fact, the position of 75 bp upstream of *murF*, where the non-capnophilic strain 21P20-nc2 had a single-nucleotide substitution, was predicted to be a promoter region by Promotech. In contrast, whether the position of 20 bp upstream of *murF* where the non-capnophilic strains 21P20-nc1 and 21P20-nc3 have single-nucleotide substitution, the 16 bp upstream of *murF* where 17P48-nc3 has a single-nucleotide substitution, and the 15 bp upstream of *murF* where 20P18-nc1 and 20P18-nc2 have a single-nucleotide substitution are regions involved in the regulation of *murF* expression has not been confirmed by bioinformatics tools or other means. Although these positions may be repressor-binding sites based on the positional relationship between the *murF* translation start site and the predicted promoter region, further investigation, including functional analysis, will be needed to confirm this hypothesis. The remaining 6 of the 25 non-capnophilic strains showed no substitutions in *murF* or surrounding sequences compared to that seen in their respective parental strains. These results suggest that the mechanism of CO_2_-dependent of 24F ST162 pneumococcal strains can be partly explained by the dysfunction of MurF; however, further investigation is required to elucidate the other molecular mechanisms involved in conferring CO_2_ dependence.

Burghout et al. reported that CO_2_-dependent MurF^V179^ strains were frequently detected in serotype 9V or 19F ST162 strains (13), whereas all pneumococcal isolates in this study were serotype 24F ST162. Serotypes 9V and 19F are covered by PCV7; thus, they are rarely isolated as the cause of IPD owing to the widespread availability of the vaccine. As recombination events leading to serotype switching occur relatively frequently in pneumococcal populations (26), the temperature- and CO_2_-dependent serotype 24F ST162 strain may have emerged by capsular switching after PCV introduction (7, 27, 28). Similar to the report by Burghout et al. that the CO_2_-dependent strain with MurF^V179^ primarily belonged to ST162 (13), all isolates we tested in this study that had MurF^V179^ and a CO_2_-dependent phenotype also belonged to ST162. Therefore, we speculate that these characteristics may be conserved within ST162. In this relation, we assume that the fact that all 28 of serotypes 24 (ST2572, ST5496, ST2754) except ST162 had MurF^A179^ and were non-capnophilic strains (data not shown), paradoxically supporting this hypothesis.

All isolates in this study were susceptible to penicillin G and resistant to trimethoprim-sulfamethoxazole, which is consistent with the results of several previous reports (5 – 7). Serotype 24F ST162 isolates that required CO_2_ for growth at 35°C failed to grow by the broth microdilution method in ambient air; therefore, the test must be retested under a CO_2_ environment to obtain antimicrobial susceptibility results. In particular, considering that serotype 24F is highly invasive (29, 30) and is often isolated from patients with IPD, antimicrobial susceptibility results are essential for appropriate antimicrobial therapy, and taking an extra day to report the results due to retesting may be a clinical problem. In addition, MIC values of macrolides tend to increase under CO_2_ conditions (31 – 33). In the ATCC 49619 data that we tested in this study, the MIC of clarithromycin was within the quality control range under 5% CO_2_ condition; however, the MICs of clindamycin and trimethoprim-sulfamethoxazole were higher than the quality control range. Comparing the antimicrobial susceptibility patterns of the CO_2_-dependent isolates under 5% CO_2_ and the non-capnophilic strains under ambient air, the interpretive categories of all antimicrobial agents did not change; however, the MICs of clarithromycin and clindamycin were slightly higher under 5% CO_2_. It is possible that CO_2_ may have an effect on the elevated MIC values of these agents, and therefore, caution should be exercised in the interpretation of antimicrobial susceptibility results for CO_2_-dependent serotype 24F ST 162 isolates, which would be performed using the broth microdilution method at 35°C under 5% CO_2_.

The present study has some limitations. First, although the test isolates were collected from several regions of Japan, they were not obtained from the nationwide survey in Japan. According to the nationwide survey data on IPD in Japan, serotype 24F ST162 strains are rarely detected. Therefore, a population-based survey must be conducted as serotype 24F ST162 strains may have increased in the past few years and should be monitored throughout Japan. In addition, as the burden of IPD has been reported to correlate with nasopharyngeal carriage rates, attention to serotype 24F ST162 strain trends in future population-based carriage surveillance studies may provide valuable information for the prevention and/or diagnosis of IPD. Second, a functional analysis of the nucleotide substitutions upstream of *murF* detected by comparison in CO_2_-dependent and non-capnophilic strains has not yet been performed and requires further investigation. Third, the changes in MurF of non-capnophilic strains obtained from CO_2_-dependent isolates with MurF^V179^ are somewhat inconsistent; hence, we were unable to fully determine the cause of the CO_2_ dependence of serotype 24F ST162 isolates in this study. To clarify the mechanism involved in the CO_2_-dependent growth of *S. pneumoniae* and its phenotypic change, further studies are needed.

In conclusion, we characterized the temperature and CO_2_ dependence and the associated molecular basis of the pneumococcal isolate serotype 24F ST162, which has been identified as an invasive serotype since the introduction of PCV13. Given that the prevalence of IPD caused by NVTs, in particular serotype 24F, has increased since the introduction of PCVs, when antimicrobial susceptibility for an IPD case cannot be determined via the broth microdilution method, which is usually performed at 35–37°C in ambient air condition, it might point toward 24F ST162 isolates as the suspected cause of IPD based on the current epidemiological information. Consequently, penicillin G may be tried as a therapeutic agent because all 10 serotype 24F ST162 isolates in this study were susceptible to penicillin G. However, as it is important to administer antimicrobial therapy based on antimicrobial susceptibility results, we expect that this report will lead to the accumulation of knowledge on CO_2_-dependent *S. pneumoniae*, standardization of antimicrobial susceptibility testing for bacteria with similar characteristics, and appropriate antimicrobial selection based on the results of such testing. Collectively, our findings highlight the necessity to continuously monitor the epidemiology of pneumococci, including serotype 24F ST162, isolated from patients with IPD.

## MATERIALS AND METHODS

### Bacterial isolates and growth conditions

We collected 10 isolates from sterile sites of Japanese children with IPD caused by serotype 24F ST162. The pneumococcal isolates were phenotypically identified using optochin susceptibility and bile solubility tests. The non-capnophilic strains were obtained by subculturing CO_2_-dependent isolates on two plates, one under 35°C 5% CO_2_ and the other under 35°C ambient air, and collecting colonies that grew at 35°C in ambient air. This procedure was repeated until non-capnophilic strains were obtained.

Furthermore, to investigate the growth conditions of the strains, we performed the drop plate method. Todd Hewitt Yeast (THY) broth was prepared by adding 0.5% Bacto Yeast Extract (Thermo Fisher Scientific, Waltham, MA, USA) to BD Bacto Todd Hewitt Broth (Becton Dickinson, Franklin Lakes, NJ, USA). For each of the isolates to be tested, emulsify half of a 10-µL loop of bacteria from an overnight trypticase soy agar with 5% sheep blood (Nippon Becton Dickinson Company, Ltd., Tokyo, Japan) in THY broth and incubated for 2 h at 35°C under 5% CO_2_. The culture medium was then centrifuged, and the supernatant was removed, resuspended in saline, and adjusted to 0.5 McFarland. A serial 10-fold dilution was prepared, and 5 µL of each bacterial suspensions was inoculated into the trypticase soy agar with 5% sheep blood and incubated for 24 h at 35°C under 5% CO_2_ or at 35°C in ambient air or at 30°C in ambient air. In this study, general culture and antimicrobial susceptibility testing of *S. pneumoniae* were performed at 35°C based on the Manual of clinical microbiology (34) and CLSI guidelines (14). In addition, referring to a previous report (13) that examined the growth conditions of a CO_2_-dependent strain with MurF^V179^, the isolates in this study were cultured at 30°C to verify whether they exhibited the same characteristics as the previous report.

### Serotyping of *S. pneumoniae*

All 10 isolates were serotyped by Quellung reactions with pneumococcal antisera (Statens Serum Institut, Copenhagen, Denmark). First, the pool sera were used to screen for serotypes, and then, the serotypes were determined with the group sera of each serotype in the pool sera that were positive. For those with subtypes, all factor sera were tested and subtyped according to keys to pneumococcal antisera supplied by the manufacturer. The subtypes of serotype 24 were determined using factor sera 24c, 24d, and 24e, and if a positive Quellung reaction was observed only in 24d, the isolate was determined to be 24F.

### Molecular analysis

Bacterial DNA was extracted from pneumococcal suspension pretreated with lysozyme at 35°C for 1 h using an automated nucleic acid extraction system, MagDEA Dx SV and magLEAD 12gC (Precision System Science Co., Ltd., Chiba, Japan).

Multilocus sequence typing (MLST) was performed to determine ST by comparing sequences obtained from our tested isolates with those in the pneumococcal MLST database, as described previously (35, 36). The seven housekeeping loci (*aroE*, *ddl*, *gdh*, *gki*, *recP*, *spi*, and *xpt*) from the *S. pneumoniae* PubMLST database (https://pubmlst.org/) were amplified.

The sequences of the *murF* gene were determined by Sanger sequencing using a SeqStudio capillary sequencer (Thermo Fisher Scientific, Waltham, MA, USA), as previously described (13). In addition, we sequenced the genes *pca* (11) and *folC* (12), which are reportedly associated with CO_2_-dependent *S. pneumoniae*. The primers used for sequencing analysis are listed in Table S3.

### Antimicrobial susceptibility testing

Antimicrobial susceptibility tests were initially performed using the broth microdilution method according to the CLSI M07-Ed11 protocol (37). However, all 10 isolates did not grow in ambient air at 35°C; therefore, they were incubated under 5% CO_2_ at 35°C. We evaluated the following agents to understand the antimicrobial sensitivity profiles of the test isolates to various antibiotics with different mechanisms of action using dry plate “Eiken” CD54 (Eiken Chemical Co., Ltd., Tokyo, Japan), a plate for antimicrobial susceptibility testing by broth microdilution method for penicillin G, cefotaxime, ceftriaxone, imipenem, clarithromycin, clindamycin, levofloxacin, vancomycin, chloramphenicol, and trimethoprim-sulfamethoxazole. MIC breakpoints were defined according to the CLSI criteria (14). In addition, selected non-capnophilic strains obtained by subculturing the CO_2_-dependent isolate were also tested using the same panel at 35°C in ambient air. The reference *S. pneumoniae* ATCC 49619 strain was used for quality control, and testing was performed under both at 35°C in ambient air and at 35°C under 5% CO_2_.

### WGS analysis

Seven selected isolates (21P19, 21P19-nc1, 21P20, 21P20-nc1, 21P20-nc2, 21P20-nc3, and 21P20-nc4) were subjected to WGS. Short- and long-read sequencing analyses were performed on the 21P20 isolate. Genomic DNA was extracted using the Mora extract kit (Kyowa Hakko Industries, Ltd., Tokyo, Japan) for short-read sequencing and the phenol-chloroform extraction method for long-read sequencing. Short- and long-read sequencing libraries were prepared using an Illumina DNA Prep (M) Tagmentation kit (Illumina, San Diego, CA, USA) and SQK-LSK110 ligation sequencing kit (Oxford Nanopore Technologies, Oxford, UK), respectively. Short- and long-read sequencing were performed using a NovaSeq 6000 platform (Illumina) with 2 × 150 bp or 2 × 250 bp paired-end sequencing and GridION (Oxford Nanopore Technologies) with flow cell R9.4.1, respectively. Hybrid assembly of Illumina short-reads and GridION long-reads was performed using Unicycler v0.4.8 (38). For isolates other than 21P20, only Illumina short-read sequencing was performed, and the resulting raw reads were assembled using SPAdes (Galaxy version 3.15.3). The assembled whole-genome sequences were annotated using Prokka (Galaxy version 1.14.6). SNP calling was performed using Snippy (Galaxy version 0.23.2) to compare CO_2_-dependent isolates and non-capnophilic strains sequences. To identify the percentage of SNPs called by Snippy, the sequences of 21P20-nc1, 21P20-nc2, 21P20-nc3, and 21P20-nc4 were aligned to the sequences of 21P20 using bwa (version 0.7.17-r1188). Next, bam files were generated using samtools (version 1.17) and visualized using IGV (version 2.4.14). Promotech (version 1.0) (39) was used to predict promoter regions. GPSC was predicted for all genomes with PathogenWatch (https://pathogen.watch/).

## Data Availability

Nucleotide sequence data for the two CO_2_-dependent *S. pneumoniae* isolates 21P19 and 21P20 and five non-capnophilic strains 21P19-nc1, 21P20-nc1, 21P20-nc2, 21P20-nc3, and 21P20-nc4 have been submitted to the DNA Data Bank of Japan Sequenced Read Archive under the accession numbers DRR498876, AP028877, DRR498877, DRR498878, DRR498879, DRR498880, DRR498881, respectively.

## SUPPLEMENTAL MATERIAL

Tables S1 to S3 spectrum.02165-23-s0001.pdf Supplemental tables.

ASM does not own the copyrights to Supplemental Material that may be linked to, or accessed through, an article. The authors have granted ASM a non-exclusive, world-wide license to publish the Supplemental Material files. Please contact the corresponding author directly for reuse.
