# Supplementary material for: Bacteriological and molecular characterization of temperature- and CO2-dependent Streptococcus pneumoniae serotype 24F ST162 isolated from Japanese children
Source: Microbiol Spectr. 2023 Oct 12;11(6):e02165-23. doi: 10.1128/spectrum.02165-23 (PMC10714769; doi:10.1128/spectrum.02165-23)
Supplement: Tables S1 to S3 — Supplemental tables. [file spectrum.02165-23-s0001.pdf]

Table S1. Comparison of $CO_2$-dependent isolate 21P20 and non-capnophilic strains, 21P20-nc4, 21P20-nc1, 21P20-nc3, and 21P20-nc2, obtained from 21P20

| Strain no. | Position | Type | Reference | Alteration | Gene | Product | Base substitution | AA change |
|---|---|---|---|---|---|---|---|---|
| 21P20-nc4 | 1547401 | snp | T | C | *murF* | UDP-N-acetylmuramoyl-tripeptide--D-alanyl-D- alanine ligase | c.T536C | p.Val179Ala |
| 21P20-nc1 and 21P20-nc3 | 643857 | snp | C | A | *rsuA_2* | Ribosomal small subunit pseudouridine synthase A | c.C301A | p.Leu101Met |
| | 663615 | snp | G | A | *lysS* | Lysine-tRNA ligase | c.G1389A | p.Met463Ile |
| | 1145094 | frameshift | — | T | *tlyA* | Hemolysin A | c.276dupT | p.Thr93fs |
| | 1547956 | snp | C | A | — | (20-bp upstream of the start codon of *murF* ) | — | — |
| 21P20-nc2 | 1548011 | snp | A | T | — | (75-bp upstream of the start codon of *murF* ) | — | — |

Table S2. Comparison of $CO_2$-dependent isolate 21P19 and non-capnophilic strain 21P19-nc1 obtained from21P19

| Position | Type | Reference | Alteration | Gene | Product | Base substitution | AA change |
|---|---|---|---|---|---|---|---|
| 57011 | snp | T | C | | | — | — |
| 63287 | snp | G | T | *yheH* | putative multidrug resistance ABC transporter ATP-binding/permease protein YheH | c.1697G>T | p.Gly566Val |
| 54849 | snp | A | G | | hypothetical protein | c.52A>G | p.Ile18Val |
| 54861 | snp | G | A | | hypothetical protein | c.64G>A | p.Asp22Asn |
| 47249 | complex | TCTT | GTTG | | hypothetical protein | c.245_248delTCTTinsGTTG | p.PheLeu82CysTrp |
| 47302 | snp | A | G | | ISL3 family transposase ISSpn14 | c.14A>G | p.His5Arg |
| 47321 | complex | GTTTC | ATTGG | | ISL3 family transposase ISSpn14 | c.33_37delGTTTCinsATTGG | p.PheHis12LeuAsp |
| 5762 | snp | C | T | | hypothetical protein | c.112G>A | p.Ala38Thr |
| 10731 | snp | C | A | | | — | — |
| 10740 | snp | C | T | | | — | — |
| 11053 | snp | T | C | | IS630 family transposase IS630-Spn1 | c.164A>G | p.Asn55Ser |
| 11072 | snp | C | T | | IS630 family transposase IS630-Spn1 | c.145G>A | p.Glu49Lys |

Table S3. List of primers used for polymerase chain reaction (PCR) and sequencing

| Target gene | Primer | Purpose | Sequence (5' to 3') |
|---|---|---|---|
| *murF* | PBmurF3 | PCR and sequencing | CTAGAGTTCTCGGACTTCAG |
| | PBmurF4 | PCR and sequencing | TAGTCCAGTATCTGTAAGAGC |
| | PBmurF5 | Sequencing | GAGATTGGCCTTCCTTACAC |
| | PBmurF13 | Sequencing | GCTCCATTGGCTGCTTTCTTCCACTCGG |
| *pca* | pca_1F_O | PCR and sequencing | AGTCCATATTCCGCACCTGAC |
| | pca_1R_O | PCR and sequencing | GCGTGACAACGATTCAGGAAG |
| | pca_1F | Sequencing | TGACATCGTCTGGTATTAGGGG |
| | pca_1R | Sequencing | GGCTTATGTTGCCCTACATGG |
| *folC* | folC_1F-O | PCR and sequencing | TGCTAGAGAAGCAAGAGAAAGTG |
| | folC_1R_O | PCR and sequencing | AGGTCGGGACGAAAATCCTG |
| | folC_1F | Sequencing | TCAAAGCCGGTCACCTTGAG |
| | folC_1R | Sequencing | GGCTCCATAGTCGGATTGGT |
